# Impact of ankylosing spondylitis on foot health and quality of life: an observational case–control study

**DOI:** 10.3389/fmed.2024.1355803

**Published:** 2024-04-26

**Authors:** Antonio Cortes-Rodríguez, Lisa Alves-Gomes, Marta Elena Losa-Iglesias, Juan Gómez-Salgado, Ricardo Becerro-de-Bengoa-Vallejo, Miguel Ángel Saavedra-García, Alonso Montiel-Luque, Daniel López-López, Ana María Jiménez-Cebrián

**Affiliations:** ^1^Research, Health and Podiatry Group, Department of Health Sciences, Faculty of Nursing and Podiatry, Universidade da Coruña, Industrial Campus of Ferrol, Ferrol, Spain; ^2^Nursing School, Nursing Research Centre (CIEnf) of the University of Minho, Portugal; Health Sciences Research Unit: Nursing (UICISA: E), Nursing School of Coimbra, Braga, Portugal; ^3^Faculty of Health Sciences, Universidad Rey Juan Carlos, Alcorcón, Spain; ^4^Departamento de Sociología, Trabajo Social y Salud Pública, Universidad de Huelva, Huelva, Spain; ^5^Programa de Posgrado de Seguridad y Salud, Universidad Espíritu Santo, Guayaquil, Ecuador; ^6^School of Nursing, Physiotherapy and Podiatry, Universidad Complutense de Madrid, Madrid, Spain; ^7^Departamento de Educación Física y Deportiva, Grupo INCIDE, Universidade da Coruña, A Coruña, Spain; ^8^Health District Costa del Sol, Department of Nursing and Podiatry, Faculty of Health Sciences, Primary Health Care Centre San Miguel (Torremolinos), Málaga, Spain; ^9^Department Nursing and Podiatry, Faculty of Health Sciences, University of Málaga, Málaga, Spain; ^10^Instituto de Investigación Biomédica de Málaga, Málaga, Spain

**Keywords:** ankylosing spondylitis, foot deformities, foot diseases, quality of life, foot

## Abstract

**Background:**

Ankylosing spondylitis (AS) is a chronic, inflammatory, and autoimmune disease. This condition primarily affects the axial skeleton and presents direct foot involvement, such as Achilles enthesitis or plantar fascia involvement.

**Objective:**

This study aimed to investigate the impact of foot health on the quality of life of individuals with AS compared to a control group without AS.

**Materials and methods:**

A sample of 112 subjects was recruited, with a mean age of 46.80 ± 10.49 years, divided into two groups: 56 individuals with AS (cases) and 56 individuals without AS (controls). Demographic data were collected, and the scores obtained in the Foot Health Status Questionnaire domains were recorded.

**Results:**

Of the participants, 27.79% (*N* = 30) were men and 73.21% (*N* = 82) were women. The mean age in the group was 46.80 ± 10.49. Significant differences (*p* < 0.05) were found in the domains of foot function, foot pain, footwear, overall foot health, general health-related physical activity, and social capacity between the AS group and the control group.

**Conclusion:**

Individuals with AS exhibited a decreased quality of life, as indicated by their Foot Health Status Questionnaire scores.

## 1 Introduction

Ankylosing spondylitis (AS), also known as radiographic axial spondyloarthritis, is a chronic, inflammatory, and autoimmune disease classified within the group of spondyloarthritis (1–3). This group of diseases shares clinical characteristics including involvement of the axial skeleton (sacroiliitis and ankylosed spine), peripheral manifestations (enthesitis, dactylitis, and lower limb arthritis), and extra-articular features (uveitis, psoriasis, bowel disease, kidney, lung, heart, skin, and bone) (4, 5).

The prevalence of AS varies worldwide, ranging from 0.1 to 1.4%; however, there is a lack of sufficient prevalence studies (6, 7). In 2013, Dean et al. concluded that significant prevalence differences existed across all continents, with higher rates in Europe and Asia and a sex ratio of 3.4:1 (male:female) (6). The influence of this condition is also noticeable across multiple dimensions of workforce engagement, spanning from a heightened reliance on support in paid employment to workforce disengagement. Additionally, individuals with AS, along with society at large, incur significant healthcare costs related to medications and healthcare service providers (8–10).

However, there are no studies assessing foot-related quality of life in AS individuals, making this study valuable in emphasizing the importance of foot evaluation.

Such issues can exert repercussions on both occupational and personal activities, akin to patterns observed within the broader populace where foot health complications are pervasive (ranging from 71 to 93%). These complications stem from multifaceted origins and hold the potential to prognosticate a decline in self-reliance, increased susceptibility, heightened vulnerability, compromised quality of life, and overall wellbeing (11–13).

AS typically starts with insidious lower back pain and morning stiffness. Its main symptoms are divided between joint-related (lower back stiffness, sacroiliitis, etc.) and extra-articular (uveitis, upper lobe lung fibrosis, etc.) manifestations. Enthesitis is common, often causing local pain. It predominantly occurs in the lower extremities, especially affecting the Achilles tendon insertions and plantar fascia (2, 14). Slouma et al. concluded that heel enthesis ultrasound lesions are frequent in spondyloarthropathies.

Given the previously unmet need for comprehensive and ongoing podiatric care in patients with AS, it is crucial to include foot-related pathologies, postural anomalies, and key comorbidities in the development of treatment plans and preventive measures. This approach aims to enhance the overall quality of life and wellbeing of individuals living with AS.

To date, the impact of foot health and quality of life on AS individuals has not been studied. In this study, we used the Foot Health Status Questionnaire (FHSQ) to analyze both foot-specific factors (pain, function, and footwear) and overall wellbeing factors (general health, physical activity, social capacity, and vigor).

This investigation aims to investigate the impact of foot health on the quality of life of individuals with AS compared to a control group without AS.

## 2 Materials and methods

### 2.1 Sample design

A descriptive observational case–control study was conducted following the Strengthening the Reporting of Observational Studies in Epidemiology guidelines (15).

A consecutive non-random sampling method was used to recruit a total of 112 participants, 56 with AS (cases group) and 56 healthy individuals (control group). AS participants were recruited from patient associations in Córdoba and Sevilla, while control group participants were recruited from the podiatry departments of the medical specialty centers Policlínica Alhaurín Torre Salud and Policlínica Lacibis.

Both cases and controls were informed in advance about their participation in a study on foot health and quality of life. All participants provided informed consent and agreed to take part in the study.

The inclusion criteria comprised being over 18 years old, having the ability to walk, and having signed informed consent. Exclusion criteria included having undergone lower limb surgeries and not being in full mental capacity.

### 2.2 Sample size calculation

To determine the required sample size for our case–control study, we used version 4.2 of the EpiData software, developed by the Health Department of the Xunta de Galicia, Spain, in collaboration with the Pan American Health Organization (PAHO-WHO) and Universidad CES, Colombia. The calculation was based on parameters including a 95% confidence level, a statistical power of 80%, an expected odds ratio of 2.0, and presumed exposure rates of 50% in case participants and 33.333% in control participants. This led to the identification of a necessary sample of 112 individuals, evenly divided between 56 cases and 56 controls.

### 2.3 Procedure

A sole investigator conducted all the measurements. Participant height, weight, and BMI were collected at the beginning of the interview and were used to match the participants from the case group with the control group. Subsequently, subjects completed the self-reported FHSQ, acknowledged as a validated instrument (16, 17).

The assessment of health-related quality of life, encompassing both general and specific foot aspects, was carried out using the validated Spanish version 1.03 (18) of the FHSQ. This questionnaire consists of three primary sections. The initial section, comprising 13 items, is divided into four specific domains evaluating foot health-related quality of life, encompassing dimensions such as foot health, foot pain, footwear, and overall foot health. This section has demonstrated high content, criterion, and construct validity (Cronbach’s *α* = 0.89–0.95), as well as noteworthy test–retest reliability (intraclass correlation coefficient of 0.74–0.92) (16). Furthermore, it has been established as the most suitable measure for evaluating health-related quality of life in populations experiencing foot pain (19).

The second section focuses on four domains relating quality of life to general health, covering physical activity, overall health, social capacity, and vigor. This section largely constitutes an adaptation of the Medical Outcomes Study 36-Item Short-Form Health Survey (20).

The third section encompasses descriptive data regarding socioeconomic status, comorbidities, satisfaction, and clinical history. Each questionnaire item uses an ordinal Likert scale with multiple response options, from which participants select the most appropriate answer. Through software analysis, scores ranging from 0 to 100 are generated for each domain, with 0 representing the worst possible outcome in foot health-related quality of life and 100 indicating the best result (18).

### 2.4 Ethical considerations

This research received approval from the Ethics Committee for Experimentation at the University of Málaga (Málaga, Spain) with the code 122-2022-H. The entire study was conducted in accordance with ethical principles outlined in the Declaration of Helsinki (21).

### 2.5 Statistical analysis

Sociodemographic data, including age, height, weight, and BMI, along with independent variables, were analyzed. These data were presented as mean and standard deviation (SD), as well as the minimum and maximum range. To assess data normality, the Kolmogorov–Smirnov test was applied, with *p*-values of >0.05 indicating normal distribution. However, all study variables yielded results of *p* < 0.05, indicating a non-normal distribution. Consequently, the Mann–Whitney *U*-test was utilized to ascertain statistically significant differences between groups. Additionally, a 95% confidence level was established for the obtained results.

All statistical analyses were conducted using SPSS v27.0.1.0 (IBM Corporation, Armonk, NY, United States).

## 3 Results

### 3.1 Descriptive data

The study encompassed a sample of 112 individuals of both genders (56 with AS and 56 healthy). Of these, 26.8% (*n* = 30) were men and 73.2% (*n* = 82) were women. The mean age of the entire sample was 57.78 years (SD: 12.78), ranging from 24 to 88 years. Table 1 illustrates that the demographic and descriptive data of the study participants did not display significant differences (*p* > 0.01). Furthermore, the study observed that the average duration of illness among individuals with AS was 12.63 years.

**Table 1:** Sociodemographic and descriptive data (Spain, 2023).

Descriptive data	Total (*n* = 112)		AS (*n* = 56)		Control (*n* = 56)		*p*-value
	Mean (SD)		Mean (SD)		Mean (SD)		
Age (years)	46.8 ± 10.5 (24–70)		47.0 ± 11.2 (24–70)		46.6 ± 9.8 (28–68)		0.683^†^
Weight (kg)	70.2 ± 14.7 (44–115)		71.2 ± 16.6 (44–115)		69.2 ± 12.7 (52–102)		0.751^†^
Height (m)	1.7 ± 0.1 (1.5–1.9)		1.7 ± 0.1 (1.5–1.9)		1.7 ± 0.8 (1.5–1.9)		0.816^†^
BMI (Kg/m^2^)	25.3 ± 4.9 (17.0–44.4)		25.8 ± 5.1 (17.0–44.4)		25 ± 4.2 (19.3–39.8)		0.520^†^
Sex M/F (%)	Male (%)	Female (%)	Male (%)	Female (%)	Male (%)	Female (%)	
	30(26.8%)	82(73.2%)	15(26.8%)	41(73.2%)	15(26.8%)	41(73.2%)	1^‡^

BMI, body mass index; M, male. F, female. In all analyses, a *p*-value of < 0.05 (with a 95% confidence interval) was considered statistically significant. ^†^U de Mann–Whitney *U*-test was applied. ^‡^Frequencies (percentages) and chi-square (*X*^2^) test were utilized.

### 3.2 Comparison of patients with and without AS

All domains exhibited a normal distribution (*p* < 0.05). The results of comparing the scores obtained for different FHSQ domains are presented in Table 2. Statistically significant differences (*p* < 0.05) were observed between the case and control groups across all specific foot domains (foot pain, foot function, foot health, and footwear), as well as within most general domains (overall health, physical activity, and social capacity). Therefore, the results indicate that the AS population presents lower quality of life concerning foot health and overall wellbeing compared to the matched healthy population. The vitality domain is the only one showing no significant differences between the groups.

**Table 2:** Comparisons of foot health status questionnaire scores (Spain, 2023).

FSHQ domains	Total (*n* = 112)	AS (*n* = 56)	Control (*n* = 56)	*p*-value
Foot pain	65.0 ± 27.8 (0–100)	51.9 ± 29.0 (0–100)	78.2 ± 19.2	**<0.001** ^†^
Foot function	68.9 ± 30.9 (0–100)	54.5 ± 32.1 (0–100)	83.3 ± 21.7 (0–100)	**<0.001** ^†^
Footwear	43.9 ± 33.1 (0–100)	34.5 ± 33.1 (0–100)	53.3 ± 30.7 (0–100)	**0.002** ^†^
General foot health	51.1 ± 31.3 (0–100)	35.8 ± 27.6 (0–100)	66.3 ± 27.3 (0–100)	**<0.001** ^†^
General health	55.4 ± 29.6 (0–100)	35.8 ± 21.6 (0–100)	75 ± 22.9 (0–100)	**<0.001** ^†^
Physical activity	68.7 ± 27.8 (0–100)	50.1 ± 23.3 (0–100)	87.4 ± 17.5 (0–100)	**<0.001** ^†^
Social capacity	64.2 ± 33.5 (0–100)	46.2 ± 32.3 (0–100)	82.1 ± 23.8 (0–100)	**<0.001** ^†^
Vigor	54.1 ± 23.7 (6.25–100)	54.7 ± 24.9 (6.25–100)	53.6 ± 22.6 (6.25–100)	0.963^†^

FHSQ, Foot Health Status Questionnaire. ^†^U de Mann–Whitney *U*-test was applied. In all analyses, a *p*-value of < 0.05 (with a 95% confidence interval) was considered statistically significant (bold).

## 4 Discussion

The aim of this study was to investigate the impact of foot health on the quality of life of individuals with AS compared to a control group without AS.

Upon evaluating the results of our study, a non-significant difference was found in the vigor domain levels between the AS group and the control group, which aligns with findings from other studies conducted in chronic diseases (22–24). However, significant differences (*p* < 0.05) were observed in the remaining domains, including foot pain, foot function, footwear, overall foot health, overall health, physical activity, and social capacity. These results are consistent with findings from other studies that assessed foot health-related quality of life using the FHSQ tool, such as a study conducted in multiple sclerosis patients (25) and fibromyalgia patients (26), both chronic conditions and degenerative conditions affecting the musculoskeletal system, such as AS. Therefore, it would be worthwhile to conduct further research to correlate these findings with the duration of AS diagnosis or to explore differences between men and women with the disease, as suggested by another study (27).

Several studies have indicated that AS has a direct impact on the foot (28–31), For instance, Koka et al. analyzed the impact of AS on the foot and found a significantly higher foot function index in individuals with AS, concluding that the foot and ankle are frequently affected in AS individuals (28). Sahli et al. assessed foot impact in spondyloarthritis patients, considering symptoms, deformity type and frequency, localization, and radiological changes, finding that 52% of patients showed foot involvement (31). Previous studies have also examined how gait is significantly altered in patients with AS (32, 33), and further research has suggested that these gait parameters might predict physical function in axial spondyloarthritis patients (34).

Considering both direct effects, such as Achilles tendon involvement, and indirect effects, such as gait changes induced by AS at the foot level, which significantly increase pain (32, 33, 35–37).

Previously, it was found that individuals with AS experienced peripheral joint involvement and severe disability, leading to a reduced quality of life (38). A recent study concluded that the quality of life in AS patients was poor and correlated with high disease activity (39). Quality of life in AS patients has been studied by various authors, and the consensus is that it is inferior to the general population, particularly in the dimensions of physical health, mental health, and social role (40). Moreover, certain authors advocate that AS treatments should be oriented toward enhancing patients’ quality of life (41).

In addition, the present study had some limitations. First, identifying the presence of comorbidities in the control group would be beneficial to strengthen the study and may help identify factors where this association does not exist, as well as the mechanisms involved. Second, the absence of blinding the evaluator to participant group assignments, and third, the geographical origin of participants, stemming from two distinct locales (Córdoba and Seville).

Finally, future research would benefit from larger sample sizes, investigation among different cultures, ethnicities, and living locations, and a random sampling approach to enhance the generalizability of findings.

## 5 Conclusion

This investigation provides further evidence that individuals with AS exhibit a decreased quality of life, as indicated by the FHSQ scores. Consequently, regular checks of the foot are crucial to improving overall foot health status and the wellbeing of individuals affected by AS.

## Data availability statement

The raw data supporting the conclusions of this article will be made available by the authors, without undue reservation.

## Ethics statement

The studies involving humans were approved by Ethics Committee for Experimentation at the University of Málaga (Málaga, Spain) with the code 122-2022-H. The studies were conducted in accordance with the local legislation and institutional requirements. The participants provided their written informed consent to participate in this study.

## Author contributions

AC-R: Conceptualization, Data curation, Formal analysis, Investigation, Methodology, Supervision, Writing – original draft, Writing – review & editing. LA-G: Conceptualization, Formal analysis, Investigation, Methodology, Supervision, Writing – original draft, Writing – review & editing. ML-l: Conceptualization, Formal analysis, Investigation, Methodology, Supervision, Writing – original draft, Writing – review & editing. JG-S: Conceptualization, Formal analysis, Investigation, Methodology, Supervision, Writing – original draft, Writing – review & editing. RB-d-B-V: Conceptualization, Formal analysis, Investigation, Methodology, Supervision, Writing – original draft, Writing – review & editing. MS-G: Conceptualization, Formal analysis, Investigation, Methodology, Supervision, Writing – original draft, Writing – review & editing. AM-L: Conceptualization, Formal analysis, Investigation, Methodology, Supervision, Writing – original draft, Writing – review & editing. DL-L: Conceptualization, Formal analysis, Investigation, Methodology, Supervision, Writing – original draft, Writing – review & editing. AJ-C: Conceptualization, Formal analysis, Investigation, Methodology, Supervision, Writing – original draft, Writing – review & editing.
